# Gambling disorder in adolescents: what do we know about this social problem and its consequences?

**DOI:** 10.1186/s13052-018-0592-8

**Published:** 2018-12-04

**Authors:** Pietro Ferrara, Giulia Franceschini, Giovanni Corsello

**Affiliations:** 1 Institute of Pediatrics, Catholic University Medical School, Rome, Italy; 2 Service of Pediatrics, Campus Bio-Medico University, Rome, Italy; 3 Institute of Pediatrics, University of Palermo, Palermo, Italy

**Keywords:** Gambling disorder, Adolescents, Consequences

## Abstract

Gambling disorder (GD) is a psychiatric condition and it is characterized by a maladaptive pattern of gambling behavior that persists despite negative consequences in major areas of life functioning. In Italy, CNR (National Research Council) underlined how over 17 million, 42.8% of the population aged 15–64 have a gambling behavior. Among them, there are over one million students, aged 15–19, equal to 44.2% of Italian students; the number of minors in Italy with GD in 2017 was 580,000, equal to 33.6%. Various psychosocial treatment models have been adapted for GD; on the other hand no drug has received regulatory approval in any jurisdiction as a specific psychopharmacological treatment for GD. Family therapy interventions for treatment of substance abuse problems have been adapted for adolescents GD. Given the increasing overall prevalence of adolescent gambling, it is imperative that Pediatricians appreciate that gambling problems can also afflict adolescents. In conclusion underage gambling appears to be associated positively with alcohol, tobacco and other substance use, as well as with other individual behaviors, therefore we need that collaborative efforts between scientific societies, government and stake holders can influence the uptake of research findings necessary to implement social policies and design effective public health intervention options. Educational-based problem gambling prevention programs are important avenues in targeting at-risk behaviors among adolescents to prevent an escalation of problematic behaviors into adulthood.

While gambling is a popular “pastime” for many individuals, it remains a significant public health worldwide problem with adverse impacts on psychological, social, familial, and/or occupational functioning. Gambling is a popular and prevalent behavior among adolescents; jointly with substance use it is viewed as a public and social health concern internationally. The early onset age of gambling is a known risk factor for developing gambling disorders (GD) later in life. GD is a psychiatric condition and it is characterized by a maladaptive pattern of gambling behavior that persists despite negative consequences in major areas of life functioning. The diagnosis of pathological gambling was renamed as GD and it was recently recategorized as a non-substance-related addiction in the fifth edition of Diagnostic and Statistical Manual of Mental Health Disorders [1, 2]. The disorder was reclassified from an “Impulse-Control Disorder Not Elsewhere Classified” to one of the “Substance-Related and Addictive Disorders” in an effort to clarify the diagnosis and treatment of GD, to increase its recognition, and to improve research efforts directed to the disorder. This change also reflects recognition of the similarities between pathological gambling behavior and addiction to substances [3].

## Criteria for problematic gambling

The definition establishes a persistent and recurrent problematic gambling behavior leading to clinically significant impairment or distress, as indicated by the individual exhibiting four (or more) of the following in a 12-month period:

- needs to gamble with increasing amounts of money in order to achieve the desired excitement.
- is restless or irritable when attempting to cut down or stop gambling.
- has made repeated unsuccessful efforts to control, cut back, or stop gambling.
- is often preoccupied with gambling (e.g., having persistent thoughts of reliving past gambling experiences, handicapping or planning the next venture, thinking of ways to get money with which to gamble).
- often gambles when feeling distressed (e.g., helpless, guilty, anxious, depressed).
- after losing money gambling, often returns another day to get even (“chasing” one’s losses).
- lies to conceal the extent of involvement with gambling.
- has jeopardized or lost a significant relationship, job, or educational or career opportunity because of gambling.
- relies on others to provide money to relieve desperate financial situations caused by gambling.

Specify current severity of GD is divided into Mild (4–5 criteria), Moderate (6–7 criteria) and Severe (8–9 criteria).

If the gambling behavior is not better explained by a manic episode, it can be categorised in episodic (meeting diagnostic criteria at more than one time point, with symptoms subsiding between periods of gambling disorder for at least several months) or persistent (experiencing continuous symptoms, to meet diagnostic criteria for multiple year) and specify if it is in early remission, after full criteria for gambling disorder were previously met, none of the criteria for gambling disorder have been met for at least 3 months but for less than 12 months, and in sustained remission, after full criteria for gambling disorder were previously met, none of the criteria for gambling disorder have been met during a period of 12 months or longer [1].

As compared with adults, adolescents have been found to have high rates of problem and pathological gambling. However, relatively few adolescents seek help for gambling problems [4].

## Prevalence

International studies have consistently shown that gambling is part of the life experiences of most young people: a recent systematic review underlines there are many countries that have never carried out studies on adolescent gambling behavior. In fact, most research on adolescent gambling has been conducted in Europe, North America and Australia. However, despite the lack of research in some countries, studies show that 0.2–12.3 % of youth meet diagnostic criteria for problem gambling across five continents (notwithstanding differences in cut-offs and timeframes among assessment instruments). It should also be noted that there are some variations in problem gambling prevalence rates that occur among different continents: in North America problem gambling prevalence rates ranged from 2.1 to 2.6 %, whereas in Oceania these rates ranged from 0.2 to 4.4 %. In Europe, problem gambling prevalence rates ranged from 0.2 to 12.3 %. Consequently, European studies showed the highest and the lowest adolescent problem gambling prevalence rate [5].

In Italy, CNR (National Research Council) underlined how over 17 million, 42.8% of the population aged 15–64 have a gambling behavior. Among them, there are over one million students, aged 15–19, equal to 44.2% of Italian students; the number of minors in Italy with GD in 2017 was 580,000, equal to 33.6% [6, 7]. CNR survey shows gaming alternatives are known by almost all adolescents: 94.8% of the interviewed boys know the scratch card, almost 90% know the lotteries, 87.5% sports bets, 86.8% slot machines, 84.1% bingo. In general, the influence of relatives and the family have been proven, but adolescents can get in touch with gambling ways from advertising on TV, 80.6%, or from online advertising 67.3%, or they come in contact in the bar and pubs, 64.8% [6].

## Comorbidities and physio-pathology

Demographic, psychological, and biological associations have been identified as predictive risk factors and processes associated with the development of GD. In particular, demographic associations - including young age (adolescents), male sex, non-white ethnic origin, low socioeconomic status, and divorced or separated parents - are general risk factors that are associated with GD [7–9]. Among other results, sports gamblers tended to be young men with high rates of addiction comorbidity, whereas slot machine gamblers tended to be older women with higher rates of psychiatric comorbidity and later onset of gambling.

GD are highly comorbid with other psychiatric disorders. The strongest evidence base relates to substance use disorders: pathological gamblers had an increased risk of having a diagnosis of alcohol misuse in their lifetimes and an increased risk of having a substance use disorder [10].

Additionally, rates of major depression, dysthymia, anxiety disorder, panic disorder, and specific phobias were each more than three times higher in gamblers, with social phobia twice as high a risk [11–13]. Studies demonstrated how mood and anxiety disorders can predict the subsequent onset of pathological gambling. With regard to substance use disorders, pathological gambling more often predicted the subsequent onset of substance use disorders than viceversa. The behaviors that characterise problematic gambling (eg, chasing losses, preoccupation with gambling, inability to stop) are impulsive in that they are often premature, poorly thought out, risky, and result in deleterious long-term outcomes. Deficits in aspects of inhibition, working memory, planning, cognitive flexibility, and time management or estimation are more common in individuals with pathological gambling [11–14]. A decreased activation in the ventrolateral prefrontal cortex was reported in problem gamblers compared with healthy controls [15].

Increasing evidence implicates multiple neurotransmitter systems (eg, dopaminergic, serotonergic, noradrenergic, opioidergic) in the pathophysiology of GD. Alterations in dopaminergic pathways might underlie the seeking of rewards (ie, gambling) that trigger the release of dopamine and produce feelings of pleasure; the dopaminergic mesolimbic pathway from the ventral tegmental area to the nucleus accumbens might be involved in pathological gambling [15–18].

## Genetic and environmental aspects

Data from twin studies suggest a genetic contribution to gambling disorders [19]. Molecular genetic techniques have been used to investigate the role of genetic factors in GD: it has identified specific allele variants of candidate genes corresponding to these neurotransmitter systems associated with GD: allele variants of polymorphisms at dopamine receptor genes, the serotonin transporter gene, and the monoamine-oxidase A gene. The genetic versus environmental contributions to pathological gambling can be estimated by comparing its concordance in identical (monozygotic) and fraternal (dizygotic) twin pairs. In a study of male twins that used the Vietnam Era Twin Registry, 12–20% of the genetic variation in risk for pathological gambling and 3–8% of the nonshared variation in risk for pathological gambling was accounted for by risk for alcohol use disorder [19].

Although many genes confer vulnerability, several environmental factors also contribute to developmental pathways of gambling disorders. The structural and situational characteristics of gambling activities (eg, accessibility to gambling, location and type of gambling establishment, size and number of prizes, and near-miss opportunities) are important factors involved in the maintenance of gambling behavior [20]. Additionally, rates of early negative childhood experiences, such as abuse and trauma, seem to be higher in individuals with GD than in social gamblers, with the severity of maltreatment being associated with the severity of gambling problems and an earlier age of gambling onset.

Gambling problems affect the functioning of family and intimate relationships as well as other family members. Impaired family relationships, emotional problems and financial difficulties are some of the most common impacts on family members of people with gambling problems.

The effects of gambling problems on intimate relationships have been divided into three distinct phases:

1. the denial phase.
2. the stress phase.
3. the exhaustion phase.

The types of impacts identified in this model suggests that the intimate relationships of people with gambling problems involve poor communication, relationship, conflict and arguments, and consideration of separation or divorce [21].

The family environments of people with gambling problems are also characterised by high levels of anger and conflict as well as low levels of clear and effective communication, less independence, less engagement in intellectual and cultural activities, a lack of commitment and support, little direct expression of feelings, and less participation in social and recreational activities. These family environments are comparable to those of people with drinking problems [22]. Moreover, the children of people with gambling problems are exposed to a range of family stressors, including financial and emotional deprivation, physical isolation, inconsistent discipline, parental neglect/abuse and rejection, poor role modelling, family conflict, and reduced security and stability [23–27]. In fact several studies indicate that the clinical pictures of enuresis and other chronic diseases have a negative impact on quality of life and result in higher scores for behavioral problems when compared to control groups [24]. A previous history of nocturnal enuresis in childhood may have an abnormal neuronal response to emotional stimuli suggests that nocturnal enuresis may affect the individual both psychologically and neurologically and may lead to excessive behavior.

Common gambling problems reported by family members include:

- the loss of household or personal money.
- arguments.
- anger and violence.
- lies and deception.
- neglect of family.
- negatively affected relationships.
- poor communication.
- confusion of family roles and responsibilities.
- the development of gambling problems or other addictions within the family [28].

Gambling problems adversely affect their families in a number of ways: while emotional difficulties, physical complaints and behavioural difficulties are common, they can be experienced and expressed quite differently, as: emotional disturbances (anger, resentment, depression and anxiety), physical complaints (headaches, gastrointestinal symptoms and chronic headaches) and behavioural difficulties (excessive drinking, smoking, over/under-eating, impulsive spending, alcohol and tobacco abuse, illegal acts) [21].

## Prevention and intervention

When gambling opportunities are made available to the public in a given jurisdiction, some individuals participate occasionally and others more frequently. Among frequent gamblers, some individuals develop problematic involvement and some do not [29]. So is important the association among demographic and social risk factors, frequency of gambling and gambling disorders. Individuals with higher intelligence, males, single individuals and those exposed to gambling environments (friends and family who gamble) and those who started to gamble at a younger age were more frequent gamblers. Excitement-seeking personality traits were also higher among more frequent gamblers. A different set of risk factors was associated with the likelihood of gambling disorder among these higher-frequency gamblers. These variables included mental health indicators, childhood maltreatment and parental gambling involvement. Among higher-frequency gamblers, individuals who smoke cigarettes, those with a diagnosis of alcohol or drug dependence or obsessive–compulsive disorder, those with higher anxiety or depression and those with higher impulsivity and antisocial personality traits were more likely to report gambling-related problems. These individuals were also more likely to report gambling on electronic gambling machines (e.g. slot machines) [29]. Moreover in another study was underlined that there are no marked differences in the health and mental health status of recreational gamblers versus non-gamblers. While it is true that having drug or alcohol problems there is the correlation to recreational gambling [30]. Discriminating between recreational and at-risk gamblers also shows the importance of social networks in relation to gambling behavior. One of the strongest discriminators of being a Problem/Pathological Gambler was the portion of friends and family who regularly gamble. Indeed, having a larger portion of friends and family who are regular gamblers is a strong discriminator of being an at-risk or problem/pathological gambler and having a lower portion of friends and family who gamble regularly is the strongest correlate of being a non-gambler [30]. Despite the progress that has been made into development of effective treatments for GD, several unresolved clinical issues exist: brief treatment as telephone-based motivational interview contact combined with a mailed self-help cognitive-behavioural therapy workbook led to good outcomes over 12-month and 24-month follow-up periods [31]. Motivational interviewing is a therapeutic style of interacting with individuals to encourage them to focus on their personal reasons for needing to address problem behaviours, as well as to voice any factors that work against change. Resolution of people’s natural ambivalence about change motivates them to take action. Various psychosocial treatment models have been adapted for GD; on the other hand no drug has received regulatory approval in any jurisdiction as a specific psychopharmacological treatment for GD. Family therapy interventions for treatment of substance abuse problems have been adapted for adolescents GD.

More specifically, preventive intervention programs may seek to identify adolescents with positive attitudes toward gambling and target parents who need to strengthen their parenting resources [32]. Prevention programs might focus on increasing student perceptions of parental knowledge. The parental knowledge is in itself dependent on the willingness of young people to disclose to their parent what they are doing and thinking [32]. The first step in prevention programs may be to stimulate parental interest in their children’s whereabouts, friend choices, and day-to-day activities. This can positively affect parent-child relations and thus child’s inclination to disclose his/her life to his/her parents. Disclosing tendencies and actual parental knowledge may facilitate the discussion on adolescents’ gambling-oriented attitudes and subsequently for decreasing gambling behavior. The effects may also have implications for policy and practice, suggesting that actions should focus on societal factors that predict family connectedness and resilience as well as on improving parenting and family functioning. For example, higher expenditure on family benefits (child benefits, child-raising allowances, and so on) may affect the way in which families deploy social and economic resources. This might in turn increase parents’ ability to protect and support young people through increased parental caring and knowledge, for example [32, 33].

Given the increasing overall prevalence of adolescent gambling, it is imperative that Pediatricians appreciate that gambling problems can also afflict adolescents. It would be appropriate to have theoretical and evidence-based programs that examine approaches, potential risk and protective factors, program structure, delivery methods and structured long-term evaluation. All these factors should be taken into consideration by future researchers in developing and implementing programs that can effectively mitigate GD among adolescent.

## Conclusions

In conclusion underage gambling appears to be associated positively with alcohol, tobacco and other substance use, as well as with other individual behaviors, therefore we need that collaborative efforts between scientific societies, government and stake holders can influence the uptake of research findings necessary to implement social policies and design effective public health intervention options. Educational-based problem gambling prevention programs are important avenues in targeting at-risk behaviors among adolescents to prevent an escalation of problematic behaviors into adulthood.
